# Pathological Assessment of the AJCC Tumor Regression Grading System After Preoperative Chemoradiotherapy for Chinese Locally Advanced Rectal Cancer

**DOI:** 10.1097/MD.0000000000002272

**Published:** 2016-01-22

**Authors:** Lu-Ning Zhang, Wei-Wei Xiao, Shao-Yan Xi, Pu-Yun OuYang, Kai-Yun You, Zhi-Fan Zeng, Pei-Rong Ding, Hui-Zhong Zhang, Zhi-Zhong Pan, Rui-Hua Xu, Yuan-Hong Gao

**Affiliations:** From the Department of Radiation Oncology (L-NZ, W-WX, P-YOY, Z-FZ, Y-HG), Department of Colorectal Surgery (Z-ZP), Department of Medical Oncology (R-HX), and Department of Pathological Oncology (S-YX, H-ZZ), Sun Yat-sen University Cancer Center, State Key Laboratory of Oncology in South China, Collaborative Innovation Center for Cancer Medicine, Guangzhou, Guangdong, China; and Department of Oncology, The Second Affiliated Hospital of Sun Yat-sen University, Guangzhou, China (K-YY).

## Abstract

We used American Joint Committee on Cancer (AJCC) Staging Manual system to assess the prognostic significance of tumor regression grading (TRG) for locally advanced rectal cancer (LARC) (T3/4 or N+) patients who were treated with preoperative chemoradiotherapy (CRT).

The 4 AJCC-TRG classifications were evaluated on surgical specimens from 295 LARC patients receiving CRT. Overall survival (OS), disease-free survival (DFS), local recurrence-free survival (LRFS), and distant metastasis-free survival (DMFS) were estimated using Kaplan–Meier method and Cox regression model.

Classifications of TRG 0, 1, 2, and 3 were found in 27.5%, 19.3%, 45.7%, and 7.5% of the resected specimens, respectively. Three-year OS was 95.5% for TRG0, 91.5% for TRG1, 84.8% for TRG2, and 85.7% for TRG3 (*P* = 0.035). Three-year DFS was 89.0% for TRG0, 74.4% for TRG1, 70.9% for TRG2, and 62% for TRG3 (*P* = 0.018). By multivariate analysis, AJCC-TRG (*P* = 0.033), residual lymph node metastasis (ypN+) (*P* < 0.001) and pretreatment CA19-9 level (*P* = 0.035) were significant predictors of OS. Pathological T category (*P* = 0.006) and nodal status (*P* < 0.001) after CRT were the most important independent prognostic factors for DFS.

AJCC-TRG is a prognostic factor for LARC patients receiving CRT, independent of pathological staging.

## Abstract 2

Supplemental Digital Content is available in the text

## INTRODUCTION

Recently, the standard management for patients with locally advanced rectal cancer (LARC) is preoperative concurrent 5-fluorouracil (5-FU) or capecitabine-based chemoradiation followed by total mesorectal excision (TME) surgery. Following chemoradiotherapy (CRT), the primary tumor and mesorectal lymph nodes may show inconsistent responses ranging from a pathological complete response (pCR), to single cells or small groups of tumor cells remaining, to residual cancer with desmoplastic response or even to minimal evidence of tumor response.^1^ Grading the histological changes is exactly the alternative method to assess treatment response, which is termed tumor regression grading (TRG). This method was first introduced by Mandard et al^2^ and thereafter developed to predict the oncological outcomes.^3–14^ Although it was reported that TRG was significantly related to the risk of developing distant metastases and to disease-free survival (DFS),^14^ it remains to be clarified whether TRG can significantly predict prognosis. Furthermore, 6 TRG systems have been reported, using 3 to 5 groups, and none of these have been demonstrated as the gold standard.

Recently, the American Joint Committee on Cancer (AJCC) Staging Manual (7th edition) TRG system is showed to be more accurate than the others in classifying the response of American rectal cancer patients to CRT.^15^ We therefore conducted this study to validate the role of published 4-tier AJCC system and to evaluate other prognostic factors in Chinese LARC patients receiving preoperative CRT.

## MATERIALS AND METHODS

### Patients and TRG

This retrospective study was approved by the Institutional Review Board at Sun Yat-Sen University Cancer Center, and individual informed consent was waived given the anonymous analysis of routine data. A total of 376 patients who underwent preoperative CRT followed by radical surgery at Sun Yat-Sen University Cancer Center between October 2004 and December 2012 were recruited. Rectal carcinoma was clinically diagnosed based on abdominal and pelvic computed tomography (CT), magnetic resonance imaging (MRI), and endorectal ultrasound (ERUS). In our cancer center, ERUS is recommended for every patient for accurate T staging. Other examinations such as complete blood cell count, liver function tests, and serum carcinoembryonic antigen (CEA) and carbohydrate antigen 19-9 (CA19-9) levels were also conducted. All of the patients had biopsy-proven rectal carcinoma. Only 316 patient's specimens could be used to determine the TRG classification. Of these, another 21 patients were excluded because they had synchronous distant metastases, another primary malignancy, or a prior history of radiotherapy to the pelvis. The remaining 295 resection specimens were examined for the first round by a pathologist (S-YX) and then reviewed by another experienced pathologist (H-ZZ) for the second round in uncertain cases. Both of them were blinded to the patients’ clinical and the existed pathological outcomes.

Pathological grading of primary tumor regression was performed semi-quantitatively by determining the amount of residual tumor cells compared with the desmoplastic response. The 4 AJCC TRG classification groups were as follows: TRG0, no residual tumor cells; TRG1, single cells or small groups of cells; TRG2, residual cancer with desmoplastic response; and TRG3, minimal evidence of tumor response.

### Treatment

All patients underwent preoperative radiotherapy with a total dose of 46 to 50 Gy in 23 to 25 fractions to the primary tumor. Details of radiotherapy have been specified previously.^16^ During radiotherapy, 249 patients received XELOX (oxaliplatin 100 mg/m^2^, d1 + capecitabine 1000 mg/m^2^ bid, po, d1–14), 38 patients were administered FOLFOX6 (oxaliplatin 85 mg/m^2^, d1 + leucovorin 400 mg/m^2^, d1 + 5-FU 400 mg/m^2^ iv, d1 followed by 2400 mg/m^2^ civ 46–48 h), and the remaining 8 patients were given Xeloda alone (capecitabine 1000 mg/m^2^ bid, po, d1–14) for poor liver or kidney function.

Surgery was performed 6 to 8 weeks after the completion of preoperative CRT. All patients underwent radical proctectomy, including low anterior resection (LAR), abdominoperineal resection (APR), and Hartmann's procedure.

Postoperative adjuvant chemotherapy was recommended for all patients, irrespective of the surgical pathological results, in accordance with National Comprehensive Cancer Network guidelines. However, only 181 patients received adjuvant chemotherapy, either XELOX or FOLFOX6, 4 weeks after surgery. The other 114 patients omitted adjuvant chemotherapy owing to postoperative complications, poor overall performance status, or refusal for no reason.

### Follow Up

Patients were examined every 3 months for the first 2 years, and every 6 months thereafter. At each follow-up visit, patients were assessed by a series of conventional examination, including physical examinations (eg, digital rectal examination), complete blood cell count, liver function test, serum CEA and CA19-9 tests, chest radiography or CT, abdominal and pelvic CT or MRI, and colonoscopy. Positron emission tomography (PET)/CT was conducted when appropriate. The last follow up was completed in December 2014.

### Statistical Analysis

The primary endpoints were OS and DFS, which were defined as the time from completion of the whole treatment to death from any cause and to the first occurrence of either local or distant progression or of death in the absence of such an event, respectively. The secondary endpoints were LRFS and DMFS. Local recurrence was defined as any recurrence within the pelvic cavity or perineum. Distant metastasis was identified as any recurrence outside of the pelvic cavity.

The balance of covariates among the TRG groups was examined using *t* tests (continuous variables), χ^2^ tests or Fisher's exact tests (categorical variables), as appropriate. Overall survival (OS), DFS, local recurrence-free survival (LRFS), and distant metastasis-free survival (DMFS) rates were estimated using the Kaplan–Meier method and the log-rank test. Multivariate analysis was performed using the Cox proportional hazards regression. Two-sided *P* < 0.05 was considered statistically significant. All statistical analyses were performed using SPSS software, version 20.

## RESULTS

### Patients

The baseline characteristics of the 295 patients were listed in Table 1. Based on ERUS and/or MRI, 29% of patients were diagnosed with clinical stage II disease, and 71% were diagnosed with clinical stage III disease. A total of 77 patients (26%) had a pCR (ypT0N0M0), 40 patients (14%) had lymphatic or venous invasion, and 35 (12%) had perineural invasion. The median time interval between CRT completion and surgery was 48 days (range; 20–84 days). A total of 172 patients (58%) underwent LAR, 109 (37%) underwent APR, and 14 (5%) underwent Hartmann's procedure. The median follow up was 36 months (range; 5–120 months). There were 12 cases (4%) of locoregional relapse, 52 cases (18%) of distant metastasis, and 42 cases (14%) of death, respectively. The 3-year OS rates was 89.1% and the 3-year DFS rates was 79.5% (Table 1).

**TABLE 1:**
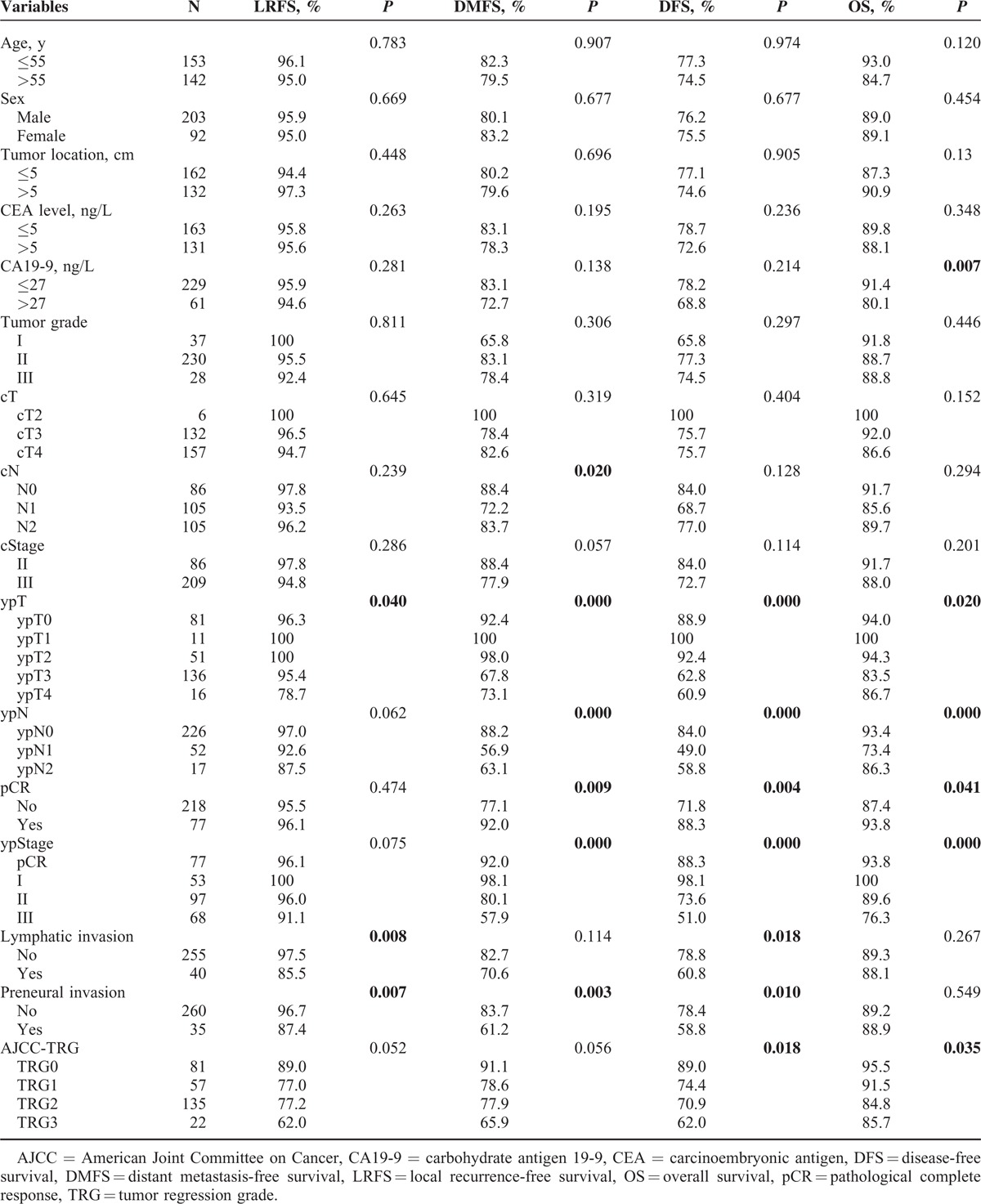
Influence of Different Variables on 3-Year Local Recurrence-Free Survival, Distant Metastasis-Free Survival, Disease-Free Survival, and Overall Survival

### TRG and the Association With Pretreatment and Postoperative Clinicopathological Factors

The associations of TRG with preoperative and postoperative factors are listed in Table 2. Overall, both pretreatment CEA levels (*P* = 0.002) and the clinical T status (*P* = 0.005) were strongly predictive of TRG. Patients with TRG 3 (59.1%) were more likely to have elevated (>5 ng/mL) pretreatment CEA levels than patients in the other TRG classes (54.1% for TRG2, 35.1% for TRG1, and 30.9% for TRG0; *P* = 0.002). Furthermore, postoperative factors, including ypT (*P* < 0.001), ypN (*P* = 0.002), lymphatic or venous invasion (*P* < 0.001), and perineural invasion (*P* < 0.001), were also significantly correlated with TRG.

**TABLE 2:**
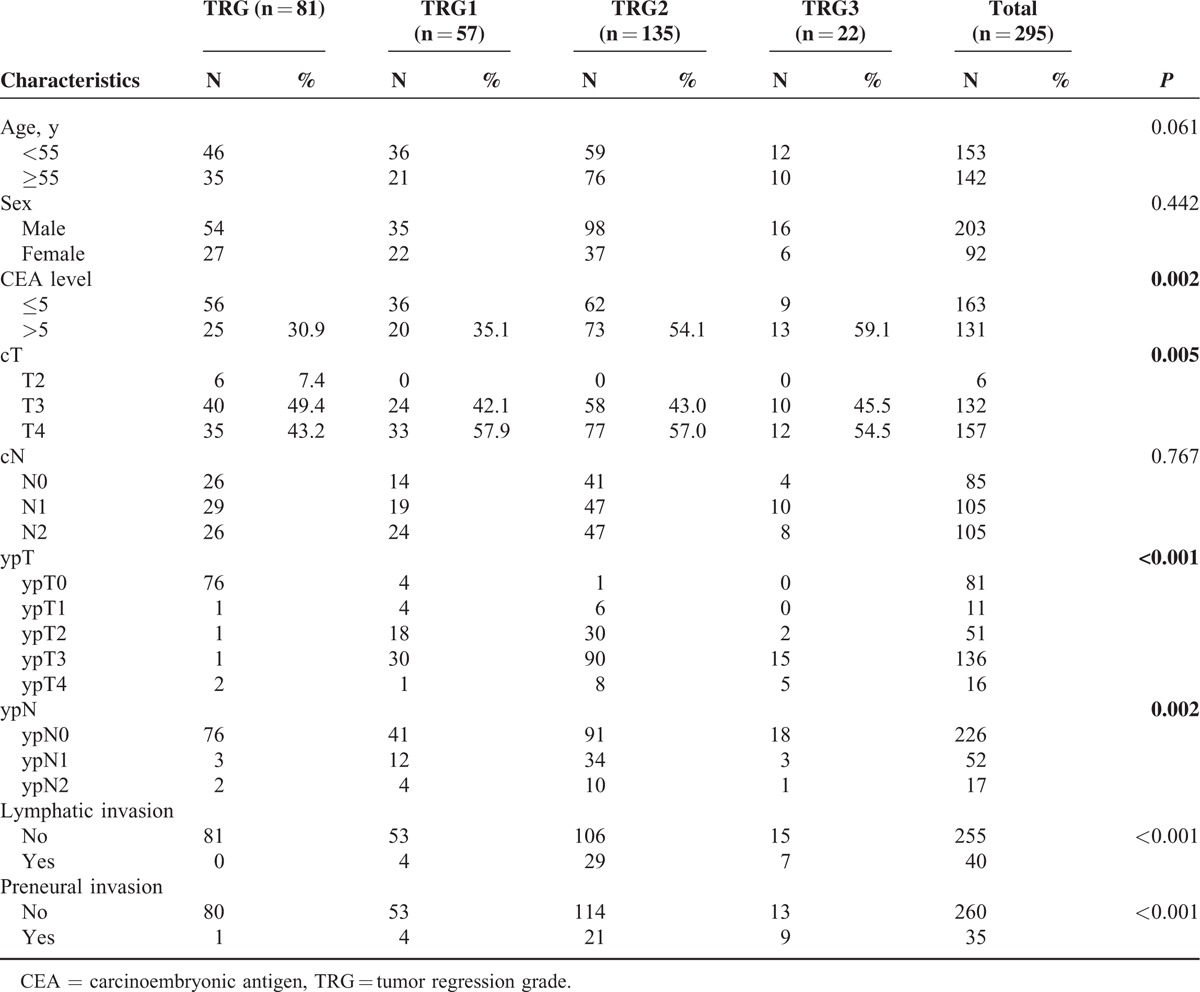
Association of TRG With Different Factors

### TRG as a Prognostic Factor for OS

**FIGURE 1:**
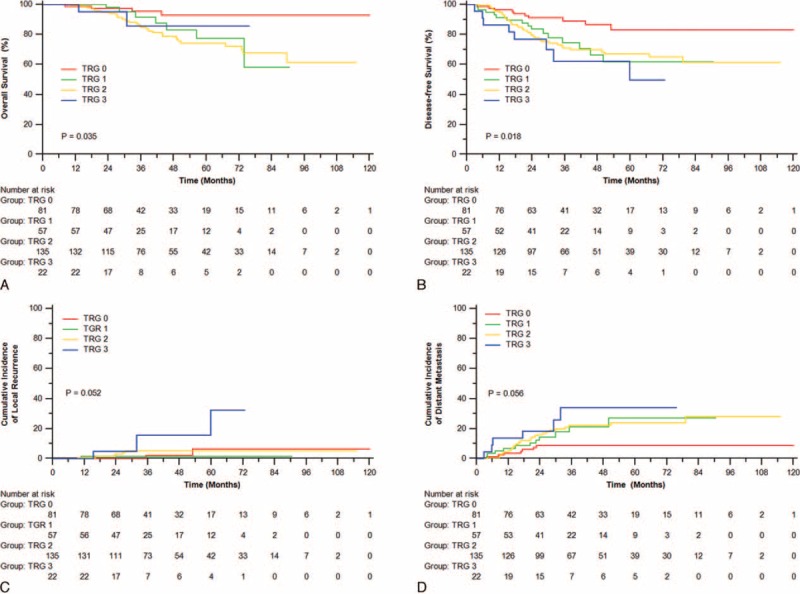
Overall survival (A), disease-free survival (B), incidence of local recurrence (C), and distant metastasis (D) of patients with different TRG classification. TRG = tumor regression grade.

In univariate analysis, TRG was significantly associated with OS (*P* = 0.035) and DFS (*P* = 0.018; Figure 1A, B). The 3-year OS rates were 95.5%, 91.5%, 84.8%, and 85.7% in patients with TRG0, TGR1, TRG2, and TRG3, respectively. The 3-year DFS rates were 89.0%, 74.4%, 70.9%, and 62.0% for patients with TRG0, TRG1, TRG2, and TRG3, respectively. Additionally, a clear trend toward less local recurrence was observed for TRG0, with a cumulative incidence of 2.3% compared with 1.8% for TRG1, 5.2% for TRG2, and 15.6% for TRG3 (*P* = 0.052, Figure 1C). A similar increasing trend was also observed for the cumulative incidence of distant metastasis (8.9%, 21.4%, 22.1%, and 34.1% for TRG0, TRG1, TRG2, and TRG3, respectively; *P* = 0.056; Figure 1D).

**FIGURE 2:**
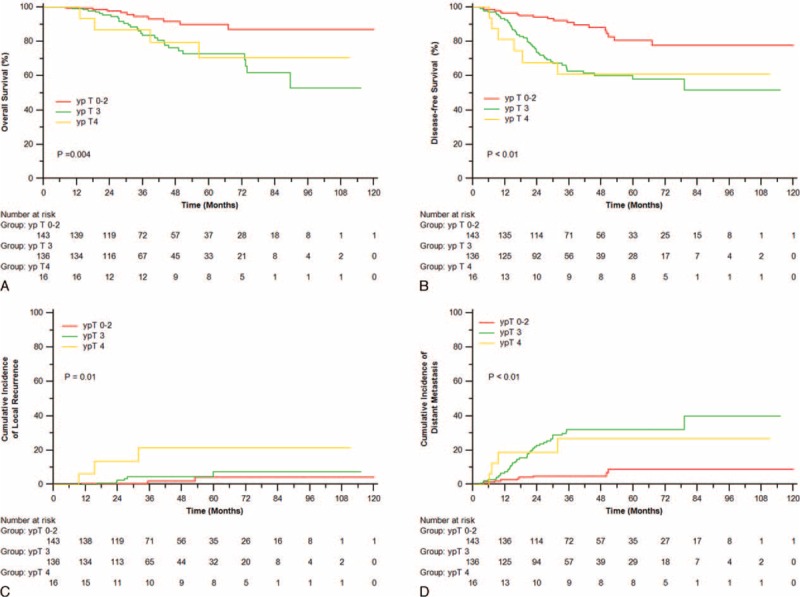
Overall survival (A), disease-free survival (B), incidence of local recurrence (C), and distant metastasis (D) of patients with different ypT.

Furthermore, we examined the prognostic significance of various clinical pathological factors (Table 1). ypT (*P* = 0.02, Figure 2A), ypN (*P* < 0.001, Supplementary Figure 1A), pCR (*P* = 0.041), ypStage (*P* < 0.001, Supplementary Figure 2A), and CA19-9 levels (*P* = 0.007) were all significantly associated with OS. DFS was significantly associated with ypT (*P* < 0.001, Figure 2B), ypN (*P* < 0.001, Supplementary Figure 1B), pCR (*P* = 0.004), ypStage (*P* < 0.001, Supplementary Figure 2B), lymphatic or venous invasion (*P* = 0.018), and perineural invasion (*P* = 0.01). LRFS was significantly correlated with ypT (*P* = 0.04, Figure 2C), lymphatic or venous invasion (*P* = 0.008) and perineural invasion (*P* = 0.007). cN (*P* = 0.02), ypT (*P* < 0.001, Figure 2D), ypN (*P* < 0.001, Supplementary Figure 1D), pCR (*P* = 0.009), ypStage (*P* < 0.001, Supplementary Figure 2D), and perineural invasion (*P* = 0.03) were all significantly associated with DMFS.

Adjusted for the above prognostic covariates, TRG was significantly associated with OS (hazard ratio [HR], 1.50; 95% confidence interval [CI], 1.03–2.19; *P* = 0.033) but not with DFS (HR, 1.177; 95% CI, 0.84–1.65; *P* = 0.34), LRFS (HR, 1.375; 95% CI, 0.64–2.97; *P* = 0.418), or DMFS (HR, 1.06; 95% CI, 0.72–1.56; *P* = 0.768). Additionally, ypT (HR, 1.31; 95% CI, 1.02–1.69; *P* = 0.006) and ypN (HR, 1.77; 95% CI, 1.23–2.54; *P* < 0.001) were associated with DFS. ypN (HR, 2.21; 95% CI, 1.50–3.27; *P* < 0.001) and CA19-9 levels (HR, 1.98; 95% CI, 1.05–3.73; *P* = 0.035) were significantly associated with OS. Only lymphatic or venous invasion was prognostically significant for LRFS (HR, 4.17; 95% CI, 1.32–15.20; *P* = 0.015). T stage (HR, 1.51; 95% CI, 1.44–1.99; *P* = 0.004) and lymph node metastasis (HR, 1.82; 95% CI, 1.26–2.64; *P* = 0.001) after preoperative CRT were prognostically significant for DMFS (Table 3).

**TABLE 3:**
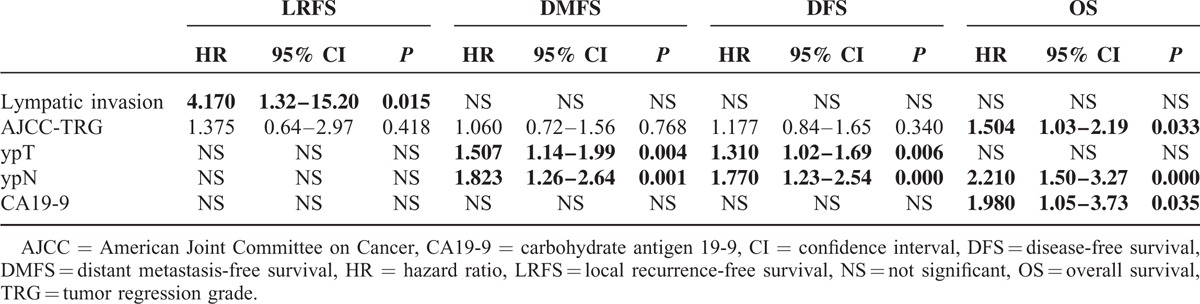
Multivariable Analysis of Different Variables on 3-Year Local Recurrence-Free Survival, Distant Metastasis-Free Survival, Disease-Free Survival, and Overall Survival

### Subgroup Analysis of Prognostic Factors for DFS

Given the strong prognostic impact of ypN+ for DFS, we investigated which factors are prognostically significant in the more favorable subgroup of patients with negative lymph nodes. As shown in Table 4, yp stage (*P* = 0.038), lymphatic or venous invasion (*P* = 0.050), and TRG (*P* = 0.026) are significantly correlated with DFS in the ypN− subgroup (Table 4).

**TABLE 4:**
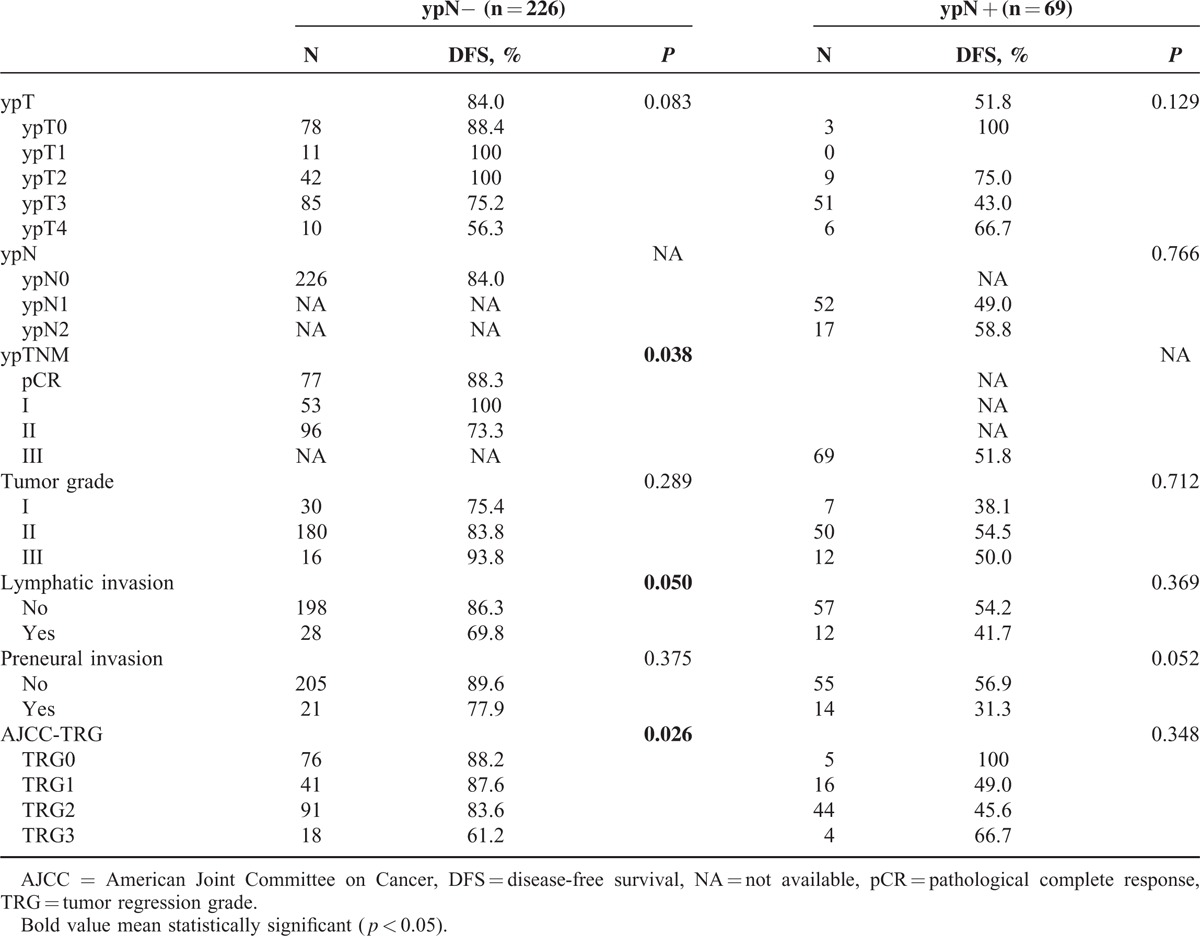
Impact of Different Clinical and Pathologic Factors on 3-Year Disease-Free Survival

## DISCUSSION

In our study, pretreatment factors, including elevated CEA level, advanced clinical T status, and postoperative factors, including ypT, ypN, lymphatic or venous invasion and perineural invasion, were strongly associated with TRG in LARC patients who underwent preoperative CRT and TME-principle surgery. Importantly, patients with higher TRG had significantly poorer OS. Subgroup analysis indicated that TRG was significantly correlated with DFS in the ypN− group.

It is known that the elevated CEA level always represents heavy tumor load which might require a higher radiation dose to achieve the same tumor response and prognosis. And advanced clinical T status are always associated with a larger tumor size which was identified as a significant factor for pCR following preoperative CRT in rectal cancer.^17,18^ Thus, the radiobiological paradigm that is dependent on tumor size to eradicate tumor cells might in part explain the observed association between clinical T category and TRG. Except the pretreatment factors, we found that some postoperative factors were associated with TRG, which is similar to the report by Claus Rödel et al.^10^ Additionally, perineural invasion was also related to TRG in the present study. This is possibly related to the autonomic pelvic nerve preservation in patients with the neural invasion.^19,20^

TRG has been showed to be an independent prognostic factor in cancers of esophagus, stomach, bladder, and head and neck.^2,21–24^ Rectal cancer patients with TRG were also found to have high incidence of distant metastasis (*P* = 0.035) and treatment failure (*P* = 0.039), but not local recurrence.^14^ But the long-term results showed significant association of TRG with DFS (*P* = 0.006) in univariate analysis rather than multivariate analysis.^10^ Notably, previous evaluations of the effect of TRG in rectal cancer were mainly restricted to non-Asian population. As the first research in the currently largest Chinese population receiving preoperative CRT, we made up for the investigation of its prognostic effect. According to the latest comparison results of various TRG staging system,^15^ the concordance index of AJCC TRG system was higher than that of the others, which indicated a better performance in predicting recurrence. Although it marginally differed from the TRG system from Memorial Sloan Kettering Cancer Center (*P* = 0.068), it is highly reasonable to use the current AJCC TRG system to prospectively collect rectal cancer staging data in our study, because of the widely accepted use of TNM staging provided by the AJCC and the need for homogeneous data.

As confirmed in previous study,^10^ we found that histopathological factors such as ypN was significantly associated with OS, DFS, and DMFS. But the accurate magnitude of ypN is highly affected by the number of retrieved lymph nodes,^25^ which can vary with age of the patient, gender, and tumor grade or site.^26^ The extent and quality of surgical resection can also undoubtedly have an impact on the node harvest. If the number of retrieved lymph nodes is insufficient, ypN is possibly inaccurate and consequently stage migration will be observed. Additionally, ypN is defined by whether a lymph node has tumor cells, regardless of the percent of tumor cells in the whole lymph node like TRG. Hence ypN may be a little vague on distinguishing the patients with slight differences in survival. In the present study, CA19-9 instead of CEA showed the tendency to predict survival of rectal cancer. This was consistent with previous finding.^27^ Unfortunately, 35% to 40% of patients with advanced colorectal cancer had increased CA19-9,^28–30^ whereas our unpublished data showed that elevated CA19-9 is observed in 17.2% of rectal cancer. So CA19-9 is not specific in rectal cancer and it may indicate tumor load with an obvious delay. Despite the variation from before to after preoperative CRT may suggest the tumor response to the treatment, this cannot provide the most direct evidence. Instead, TRG is an attempt to directly stratify the primary tumor response to chemoradiation and has been demonstrated to be associated with survival of rectal cancer, independent of other prognostic factors such as ypT and ypN.^14^ But the evaluation of TRG highly depends on the pathologist and the percent of tumor mass replaced by fibrosis or residual tumor cells in some cases are hard to exactly determine the right TRG staging category. As the reported TRG systems vary from 3 to 5 groups, there is no gold standard up to date. In addition, TRG focuses on the evaluation of primary tumor without consideration of lymph nodes. So it is appropriate to combine TRG with other prognoses such as ypN and CA19-9 to achieve increased prognostic value.

The main limitation of this study is that the 2 pathologists did not evaluate TRG from the resection specimens independently, which may lower the accuracy of TRG. But this reviewing process was actually the same as the 1 in clinical reality. Additionally, chemotherapy regimens were rarely uniform due to the retrospective design, although patients were derived from a single center to achieve homogeneous data, and clinicopathological and survival data were verified by review of individual patient record.

## CONCLUSION

AJCC-TRG is an important prognostic factor, independent of pathological staging, for LARC receiving preoperative CRT and radical resection. Thus, TRG may improve the sensitivity and specificity in predicting prognosis and may help to select subgroups of patients who might benefit from additional therapy if implemented in pathological reports.

## Supplementary Material

Supplemental Digital Content
