# Supplementary material for: Pathological Assessment of the AJCC Tumor Regression Grading System After Preoperative Chemoradiotherapy for Chinese Locally Advanced Rectal Cancer
Source: Medicine (Baltimore). 2016 Jan 22;95(3):e2272. doi: 10.1097/MD.0000000000002272 (PMC4998237; doi:10.1097/MD.0000000000002272)
Supplement: Supplemental Digital Content [file medi-95-e2272-s001.doc]

**
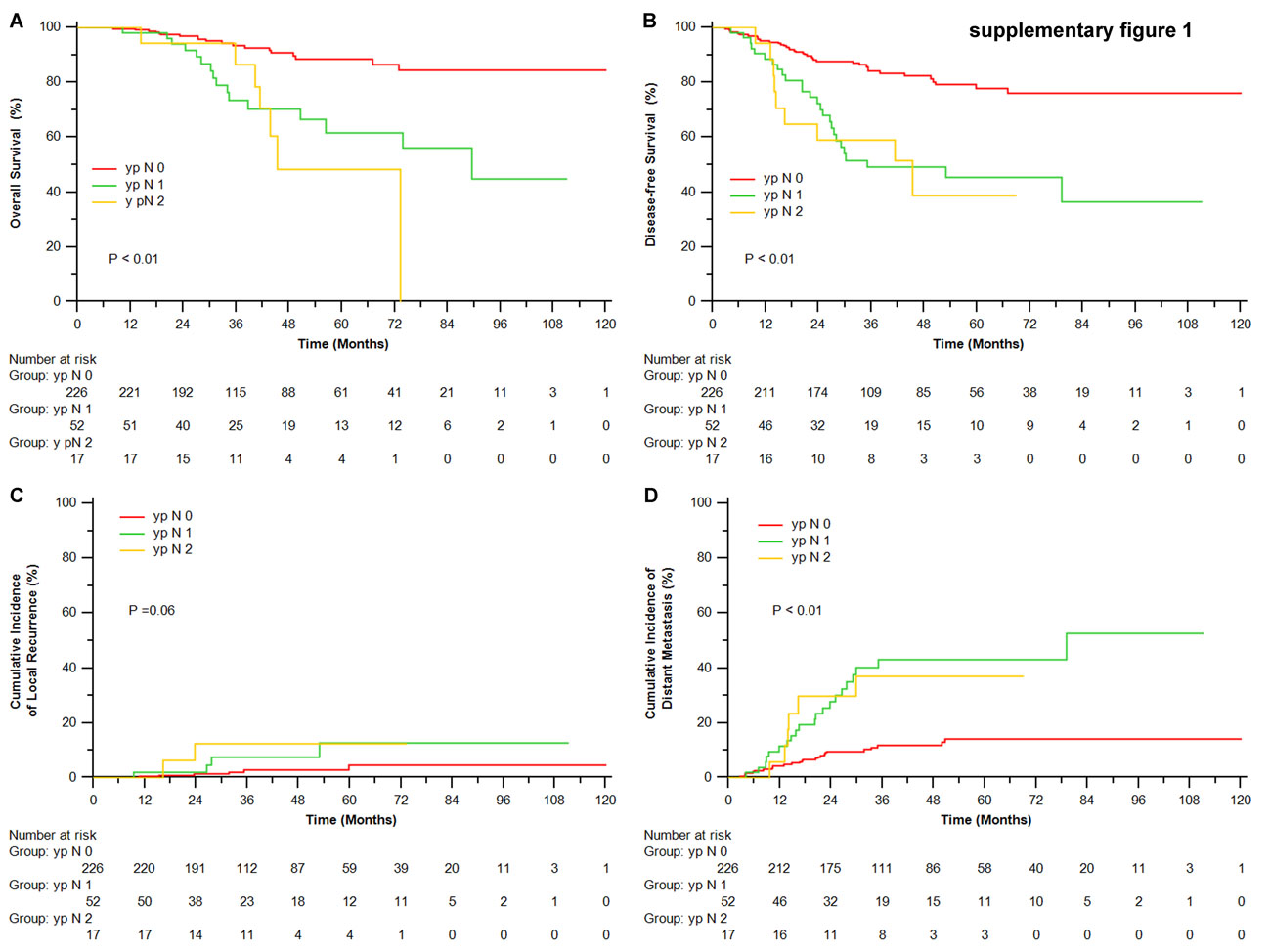
**

**Supplementary Figure 1.** Overall survival (A), disease-free survival (B), incidence of local recurrence (C) and distant metastasis (D) of patients with different ypN.

**
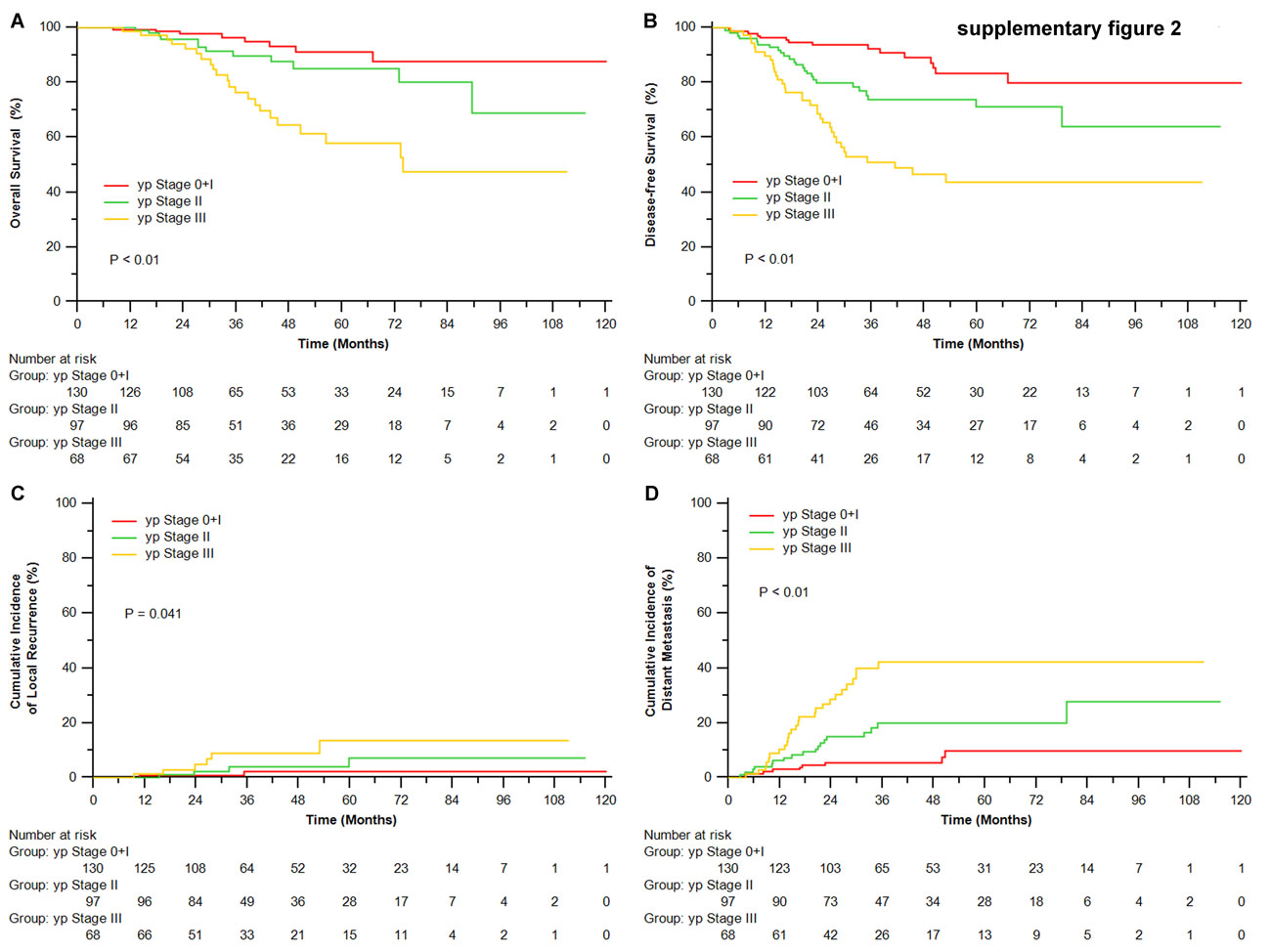
**

**Supplementary Figure 2.** Overall survival (A), disease-free survival (B), incidence of local recurrence (C) and distant metastasis (D) of patients with different ypStage.
